# Mortality in Atlanta

**Published:** 1884-10-20

**Authors:** 


### MORTALITY IN ATLANTA.

The Board of Health, in a Report for 1883, states that there were one thousand and eighty-seven deaths in the city during the year.

The greatest mortality was during the months of May, June, July and August.

Of the white population, numbering thirty thousand, there were four hundred and forty-three deaths. Of the negro population, numbering twenty thousand, there were six hundred and forty-four deaths. The total annual death rate was twenty-one and seventy-four hundredths per thousand. Percentage of mortality among the whites, fourteen and seventy-six hundredths; among the blacks, thirty-two and twenty hundredths.

The greatest mortality was in the month of July, the smallest in February.

Of the whole number of deaths, forty-nine and three hundredths per cent. occurred among children under the age of five years.

